# Superficial and peripheral dose in compensator‐based FFF beam IMRT

**DOI:** 10.1002/acm2.12018

**Published:** 2016-12-21

**Authors:** Daniel G. Zhang, Vladimir Feygelman, Eduardo G. Moros, Kujtim Latifi, Sarah Hoffe, Jessica Frakes, Geoffrey G. Zhang

**Affiliations:** ^1^ Department of Chemistry University of California Berkeley CA USA; ^2^ Radiation Oncology Moffitt Cancer Center Tampa FL USA

**Keywords:** compensator‐based IMRT, flattening filter‐free, Monte Carlo, superficial dose

## Abstract

Flattening filter‐free (FFF) beams produce higher dose rates. Combined with compensator‐based intensity modulated radiotherapy (IMRT) techniques, the dose delivery for each beam can be much shorter compared to the flattened beam MLC‐based or flattened beam compensator‐based IMRT. This ‘snap shot’ IMRT delivery is beneficial to patients for tumor motion management. Due to softer energy, superficial doses in FFF beam treatment are usually higher than those from flattened beams. Due to no flattening filter, thus less photon scattering, peripheral doses are usually lower in FFF beam treatment. However, in compensator‐based IMRT using FFF beams, the compensator is in the beam pathway. Does it introduce beam hardening effects and scattering such that the superficial dose is lower and peripheral dose is higher compared to FFF beam MLC‐based IMRT? This study applied Monte Carlo techniques to investigate the superficial and peripheral doses in compensator‐based IMRT using FFF beams and compared it to the MLC‐based IMRT using FFF beams and flattened beams. Besides varying thicknesses of brass slabs to simulate varying thicknesses of compensators, a simple cone‐shaped compensator was simulated to mimic a clinical application. The dose distribution in water phantom by the cone‐shaped compensator was then simulated by multiple MLC‐defined FFF and flattened beams with varying apertures. After normalization to the maximum dose, D_max_, the superficial and peripheral doses were compared between the FFF beam compensator‐based IMRT and FFF/flattened beam MLC‐based IMRT. The superficial dose at the central 0.5 mm depth was about 1% (of D_max_) lower in the compensator‐based 6 MV FFF (6FFF) IMRT compared to the MLC‐based 6FFF IMRT, and about 8% higher than the flattened 6 MV MLC‐based IMRT dose. At 8 cm off‐axis at depth of central maximum dose, d_max_, the peripheral dose between the 6FFF and flattened 6 MV MLC demonstrated similar doses, while the compensator dose was about 1% (of D_max_) higher. Compensators reduce the superficial doses slightly compared to open FFF beams, but increases the peripheral doses due to scatter in the compensator.

## 1 Introduction

Historically, raw x‐ray beams produced at the accelerator target were modified by an interceding flattening filter, rendering cross‐beam profiles which were fairly flat over the clinically applicable range of depths. This was done primarily for the ease of manual/forward treatment planning. However, with the advent of inverse planning techniques, the radiation beams are no longer required to be flat, and accelerators with flattening filter‐free (FFF) beams became available. The major advantage of the FFF beams is higher dose rates, leading to potentially shorter delivery times.^1^, ^2^ Because of the absence of the dominant component responsible for scatter — the flattening filter — peripheral doses are usually lower with FFF beams.^2^, ^3^

Compensator‐based intensity modulated radiotherapy (IMRT) has been applied clinically with accelerators that are not equipped with multi‐leaf collimators (MLC).^4^ The intensity modulation is accomplished by a compensator of varying thicknesses, usually made of brass using a computerized milling machine. The intensity modulation resolution in compensator‐based IMRT plans is the same as it is in MLC‐based IMRT plans. In treatment planning, an optimized ideal beam fluence map is converted into the two‐dimensional compensator thickness matrix for delivery. Even with accelerators equipped with MLC, compensator‐based IMRT technique has been used clinically^5^ because of shorter delivery times^6^, ^7^ and fewer monitor units.^8^

Upon combining FFF beams with compensator IMRT techniques, the beam‐on time can be reduced compared to the MLC‐based or compensator‐based IMRT with conventional beams, with each beam only taking a few seconds to deliver,^9^ which makes potential delivery of a breath‐hold treatment for each IMRT beam feasible. Such a possible type of ‘snap shot’ IMRT delivery would be beneficial to patients whose tumors show significant respiratory‐associated motion. This breath‐hold motion management strategy would decrease treatment times for patients who are often elderly and positioned with their arms uncomfortably overhead in a customized full body immobilization device. This very short beam‐on time advantage makes breath‐hold treatments feasible for the patients who otherwise would not be candidates for this motion management technique.

Brass compensators are commercially available so that clinics do not need to have milling machines to make them. Along with advantages, there are also some disadvantages for both the FFF beams and compensator‐based IMRT techniques. The compensators require additional time and cost to be manufactured, require the therapists to enter the room between the fields, and usually have limited modulation range.^6^ Due to the lower average energy, superficial doses in FFF beam treatment are usually higher than those from flattened beams.^10^, ^11^, ^12^ Superficial dose, defined as dose at shallow depth inside treatment field, is often a concern of patient's skin reaction to radiotherapy.^13^ In compensator‐based IMRT with FFF beams, a compensator in the beam pathway may serve simultaneously as a beam hardening filter and a scatterer. This complex interaction may affect both the superficial dose^14^ and whole‐body peripheral dose. Peripheral dose means the dose outside the treatment field. This study applied Monte Carlo techniques to investigate the superficial and peripheral doses in compensator‐based IMRT using FFF beams, and compared them to MLC‐based IMRT with both FFF and flattened beams.

## 2 Materials and methods

BEAMnrc^15^ (version V4r2.3.2), a radiation therapy Monte Carlo (MC) simulation package was used. Published phase space files for 6 MV FFF and flattened beams, generated using the actual geometry of the TrueBeam accelerator (Varian Medical System, Palo Alto, CA, USA) above the collimator moving jaws,^16^ were used as the input radiation source to BEAMnrc. Those phase space files were validated with measurements^17^ and successfully used in previous studies.^9^, ^18^, ^19^ Water phantom was simulated using DOSXYZnrc for dose distribution calculations.^20^

**Figure 1:**
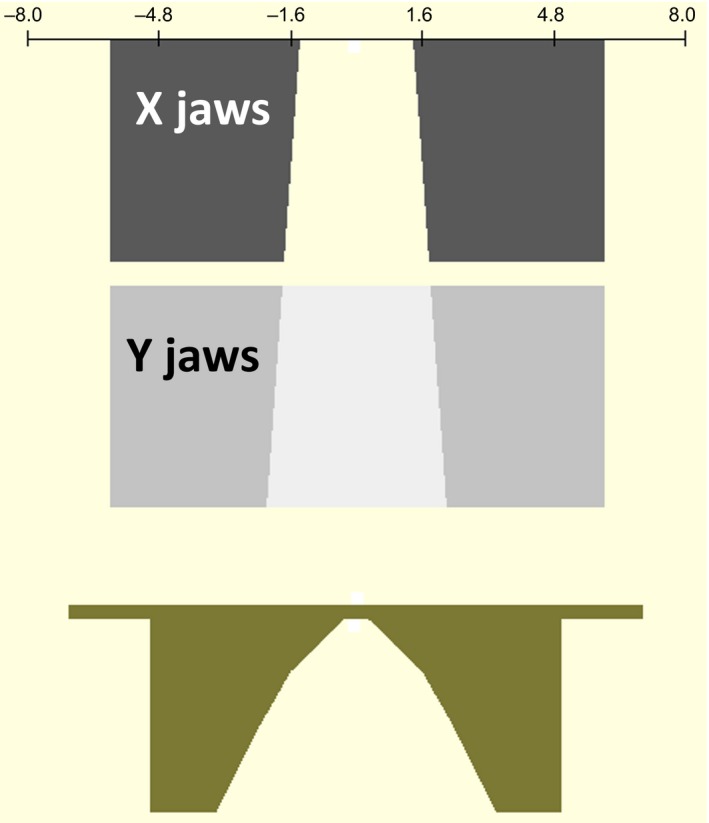
Compensator simulation setup. The collimator jaws setting was a square field of 10 × 10 cm^2^ at a distance to the source of 100 cm.

As commercial compensators for photon beam treatments are made of brass, to investigate the beam hardening effects, varying thicknesses of brass slabs in the 6 MV FFF beam were simulated, and the BEAMDP program from the BEAMnrc package was used to analyze the resulting phase space files. In addition, a simple brass cone‐shaped compensator was simulated (Fig. 1). The collimator Y jaws are turned 90° in the figure to demonstrate the geometric shape. The minimum thickness of the compensator was 0.5 cm and the maximum 7.3 cm.

Multiple MLC openings were also simulated with the 6 MV flattened and FFF beams. Dose distributions of the multiple openings in the water phantom were added with varying weightings for the 6 MV flattened and FFF beams, respectively, to match the cone‐shaped compensator dose distribution. The MLC opening sizes and the weightings were determined on trial and error basis. The source to surface distance (SSD) was 90 cm, while the distance between the bottom of the compensator and phantom surface was 25 cm. The collimator jaw settings were kept constant at 10 × 10 cm^2^ at 100 cm. The specific modalities investigated were 6 MV FFF (6FFF) compensator‐based IMRT, 6FFF MLC‐based IMRT, and 6 MV flattened MLC‐based IMRT. After the dose distributions were optimized and matched, the superficial dose (defined at the depth of 0.05 cm) along the central axis, and peripheral doses outside the field at the depth of central axis maximum dose (d_max_) were compared.

In the cone‐shaped compensator simulation, 780 million initial histories in the published phase space files were used to generate a phase space file right below the compensator, with around 10 million particles. For the dose distribution calculation, the dose grid size in the water phantom was 0.3 × 0.3 × 0.1 cm^3^, with 0.1 cm in the *z*‐direction (depth), with the finer resolution benefiting superficial dose calculations.

## 3 Results

**Figure 2:**
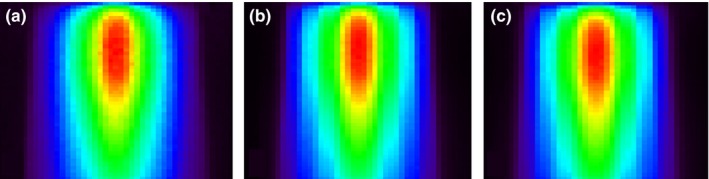
Simulated dose distributions. (a) The cone‐shaped compensator with 6FFF beam, (b) weighted summation of five various MLC openings with 6FFF, and (c) weighted summation of five various MLC openings with 6 MV flattened beams.

Figure 2 shows the simulated dose distributions of (1) the cone‐shaped compensator with 6FFF beam, (2) weighted summation of five various MLC openings with 6FFF, and (3) weighted summation of five various MLC openings with a 6 MV flattened beam. The MLC openings were the same between the FFF and flattened beams, but the relative weights differed to match the cone‐shaped compensator dose distribution.

**Figure 3:**
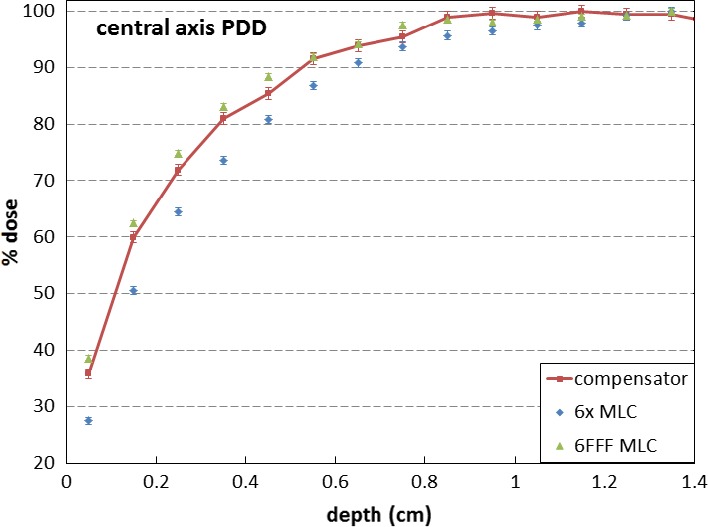
Central axis PDD comparison.

After the distributions were adjusted and matched, central axis percentage depth dose (PDD) data were extracted. The PDD data were normalized to the maximum dose along the central axis, D_max_. Figure 3 shows the central axis PDD comparison among the three modalities. The relative central superficial doses were 35.7 ± 1.0% for the compensator, 38.2 ± 0.5% for the 6FFF MLC, and 27.8 ± 0.7% for the 6 MV flattened MLC.

**Figure 4:**
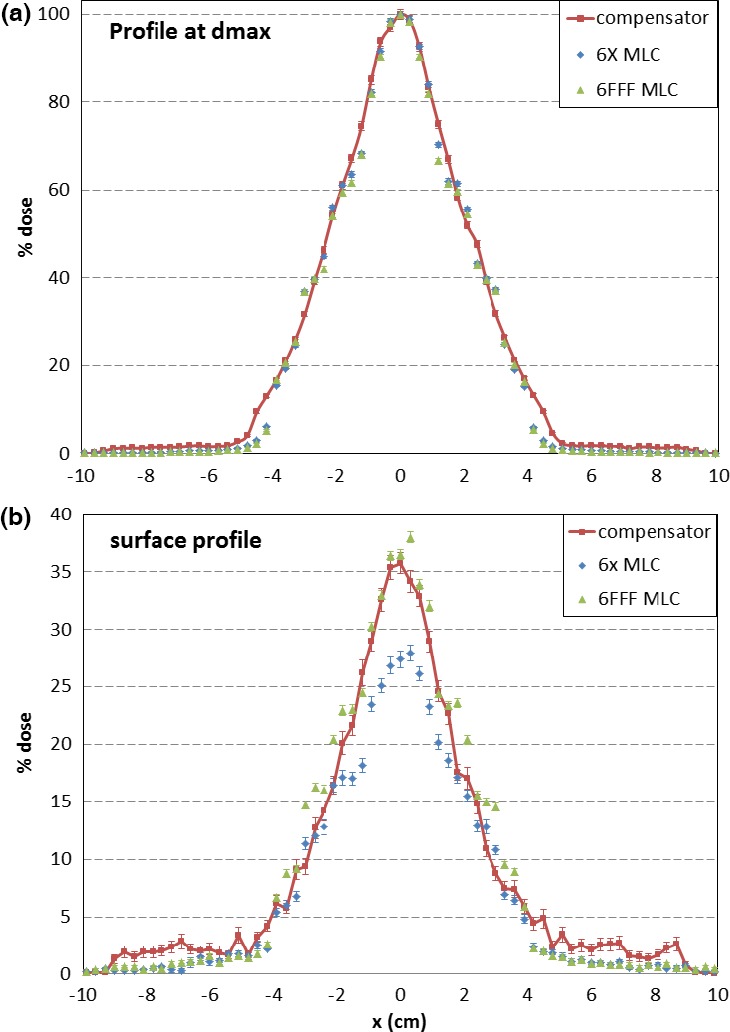
Profile comparison at (a) d_max_ and (b) superficial depth (d = 0.5 mm).

To investigate the peripheral dose difference, dose profiles at the depth of maximum dose, d_max_ (1.2 cm) and at the superficial depth (0.05 cm) were extracted. All profiles were normalized to the corresponding central axis D_max_. Figure 4 shows the profile comparison at d_max_ and at the superficial depth. At 8 cm off‐axis (about 3.5 cm outside the field) at 0.05 cm depth, the dose for the compensator‐based IMRT was 1.82 ± 0.68%, while the dose for the flattened beam MLC‐based IMRT was 0.63 ± 0.22%, and the dose for the FFF beam MLC‐based IMRT was 0.53 ± 0.15%. At depth = 1.2 cm, they were 1.34 ± 0.18%, 0.35 ± 0.05%, and 0.42 ± 0.04%, respectively. Thus, the FFF beam compensator‐based IMRT demonstrated about 1% (relative to D_max_) higher peripheral dose compared to either FFF or flattened beam MLC‐based IMRT. As the jaws setting was kept the same, the peripheral dose difference was believed to be caused by the scattering from the compensator.

**Figure 5:**
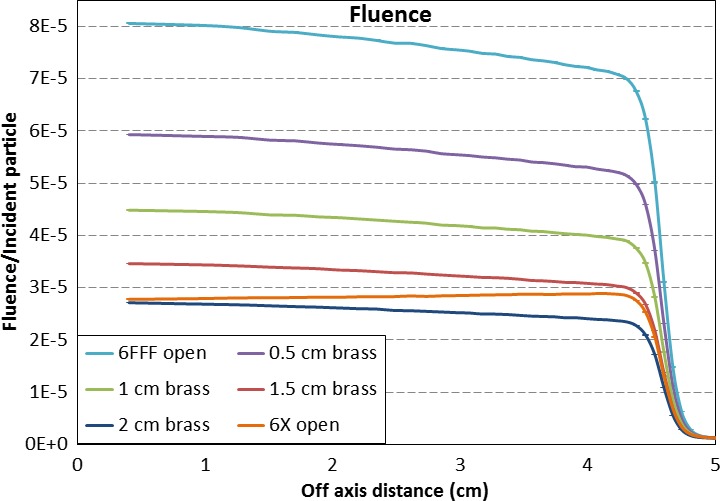
Fluence comparison of varying thicknesses of brass slabs and open FFF and flattened beams at phantom surface.

To understand why the surface dose behaves differently among the three modalities, phase space files generated with the 6FFF beams with varying thicknesses of brass slabs at the compensator location in the beam path were compared with the ones of 6FFF and flattened open beams. Figure 5 compares fluence at the phantom surface. Even with a 0.5 cm brass slab in the beam path, the fluence was still about twice as high as in the flattened beam, indicating that the effective dose rate in the compensator‐based IMRT using 6FFF beams could be twice as high as in an unmodulated 6 MV flattened beam (i.e., 3D conformal treatment). With a brass slab thickness between 1.5 and 2 cm, the dose rate would become approximately the same as in the open flattened beam.

**Figure 6:**
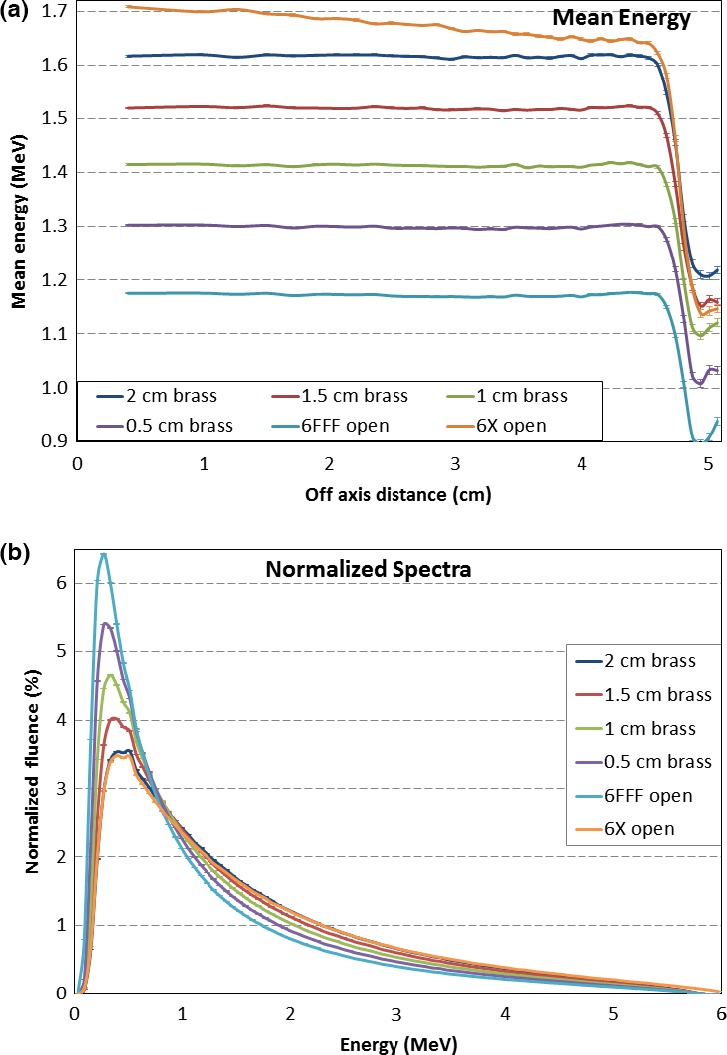
(a) Mean energy distribution comparison and (b) normalized spectra comparison for 6 MV FFF beams with various thicknesses of brass slabs in beam path. The 6 MV FFF (labeled 6FFF open in the figure) and flattened open (6X open in the figure) beams are also shown in the figure for references.

Figure 6(a) shows the mean energy as a function of off‐axis distance at the phantom surface. Beam hardening effects can be easily noticed: the thicker the brass slab, the higher the mean energy. Because flat brass slabs were used, the mean energies in the FFF configuration were essentially flat across the field, while the mean energy of the 6 MV flattened beam, as expected, was higher at the central axis.

The beam hardening effect can also be seen in Fig. 6(b), which shows the normalized spectra comparison among the various beams from Fig. 6(a). The spectra were normalized so that the area under each curve (i.e., the total fluence) was the same, set at 100%. After the normalization, the low energy fluence was decreasing in the order of FFF open, 0.5 cm, 1 cm, 1.5 cm, 2 cm brass slabs, and 6 MV flattened open beam. In the same order, the high energy fluence was increasing. The normalized spectra for the FFF beam with a 2 cm brass slab and the flattened open beam were similar.

## 4 Discussion

The main advantage of the compensator‐based IMRT using FFF beams would be the shorter per‐beam delivery time, enabling the voluntary breath‐holding motion management technique feasible for a larger subset of patients.^21^ The volumetric modulated arc therapy (VMAT) usually takes a shorter overall treatment time,^22^ but each arc is relatively long, making compensator‐based IMRT with FFF beams more practical for voluntary breath‐hold treatment delivery. As SBRT treatment strategies are being increasingly used in the treatment of patients with lung, liver, and pancreas cancers, further clinical prospective evaluation of these techniques may be warranted.

It is well known that the lower average energy in an open FFF beam results in a higher superficial dose compared to a similar flattened beam.^10^, ^11^, ^12^ The compensator could harden the FFF beam, depending on the thickness. Even with a 0.5 cm thick brass slab, the mean energy of the 6FFF beam changes from 1.17 MeV to 1.31 MeV (Fig. 6). Because of this energy difference, the 0.5 cm brass in the compensator‐based IMRT made the superficial dose slightly lower than the FFF MLC‐based IMRT (Fig. 3). With a 2 cm brass slab, the mean energy of the 6FFF beam was still lower than that of the flattened beam (Fig. 6). The thickness of material in an area of a clinical compensator projecting on the target is expected to be by far not thick enough to harden the beam to match the flattened beam. Thus, in practice, when a FFF beam with a compensator is used for IMRT, the superficial dose in the treatment area may be higher than when using flattened beam with MLC, and slightly lower than that using FFF beam with MLC.

Based on the simulation results in this study, the peripheral dose was similar between the FFF and flattened beams in MLC‐based IMRT. When combining the FFF beam with a compensator, the slightly higher peripheral dose is attributed to scatter coming from the compensator.

Only the peripheral dose near the field edge (~3 cm out of field) was analyzed in this study. The difference in those peripheral doses is mostly due to scatter. Based on the analysis in this study, the peripheral dose difference between the MLC‐based FFF and flattened beams was not significant due to similar scattered components, while the compensator‐based IMRT showed higher peripheral dose due to additional scatter from the compensator. Peripheral dose farther away from the field edge is dominated by accelerator head leakage and was not studied. Generally such dose is simply proportional to the total monitor units.

Clinical cases vary in structure size and shape, and the cone‐shaped compensator applied in this study cannot fulfill the complicate. However, the superficial dose and peripheral doses should behave similarly. Clinically, majority of the compensators are cone‐shaped but with irregular opening shapes. The BEAMnrc simulation package does not have a component module that can simulate a complicated compensator. So a real clinical compensator case cannot be simulated with the current BEAMnrc package. The simple cone‐shaped compensator was simulated using the component module BLOCK multiple times consecutively in this study.

## 5 Conclusions

High dose rate delivery in FFF beams combined with solid compensators makes breath‐hold IMRT treatments more practical than with other techniques. The beam hardening effect makes the mean energy in the compensator fields slightly higher than with open FFF beams but still below the open flattened beam. Consequently, the superficial dose is slightly lower than for the open FFF beams, but higher than for the open flattened beams. Because of the additional scatter in the compensator, peripheral dose near the field edge in compensator‐based IMRT is higher than MLC‐based IMRT using either FFF beams or flattened beams.

## Conflict of Interest

The authors declare that there is no conflict of interest.
